# Supplementary material for: Empirical examination of the replicability of associations between brain structure and psychological variables
Source: eLife. 2019 Mar 13;8:e43464. doi: 10.7554/eLife.43464 (PMC6483597; doi:10.7554/eLife.43464)
Supplement: Supplementary file 1. — Distribution of the raw phenotypical and psychological scores in the whole sample. [file elife-43464-supp1.docx]

**Supplementary file 1. Participants characteristics.** Distribution of the raw phenotypical and psychological scores in the whole sample.

| **Healthy sample** | **Participants** (n = 466 ; 153 male) |
| --- | --- |
| Age (years)  n-total = 466 | 48.34 $\pm$19.2 (18 , 85) |
| BMI (kg/m^2^)  n-total = 466 | 27.6 $\pm$5.8 (16 , 56) |
| Education (years)  n_total = 466 | 15.5 $\pm$2.25 (10 , 24) |
| Card Sorting (Free sorting)  n-total = 259 | 37.8 $\pm$10.8 (8 , 63) |
| Card Sorting (Sort recognition)  n-total = 258 | 35.5 $\pm$12.37 (8 , 63) |
| Card Sorting (sort recognition versus free sort)  n-total = 257 | -0.76 $\pm$2.1 (-7 , 4) |
| Total IQ (sum of t-scores)  n-total = 466 | 204.1 $\pm$28.7 (95 , 289) |
| Perceptual IQ (sum of t-scores)  n-total = 466 | 100.1 $\pm$17.2 (54 , 170) |
| Vocabulary IQ (sum of t-scores)  n-total = 466 | 104.3 $\pm$15.68 (21 , 149) |
| ANT (Alert) (msec)  n-total = 455 | 34.4 $\pm$31 (-88,145) |
| ANT (Orient) (msec)  n-total = 449 | 19.4 $\pm$20.2 (-44 , 90) |
| TMT (Visual scanning) (sec)  n-total = 457 | 20.5 $\pm$5 (10 , 37) |
| TMT (number sequencing) (sec)  n-total = 452 | 30.5 $\pm$10.6 (11 , 67) |
| TMT (Letter sequencing) (sec)  n-total = 452 | 30.1 $\pm$10.8 (11 , 80) |
| TMT (number-letter switching) (sec)  n-total = 447 | 77.5 $\pm$29.7 (25 , 188) |
| TMT (motor speed) (sec)  n-total = 456 | 26.3 $\pm$8.9 (2 , 58) |
| CWI (color naming) (sec)  n-total = 455 | 29 $\pm$5.6 (17 , 46) |
| CWI (word reading) (sec)  n-total = 450 | 21.5 $\pm$4 (12 ,34) |
| CWI (interference) (sec)  n-total = 449 | 54.8 $\pm$12.7 (26 , 99) |
| CWI (Inhibition/switching) (sec)  n-total = 454 | 62 $\pm$16.9 (32 , 140) |
| Verbal Fluency (letter)  n-total = 453 | 40 $\pm$12 (9 ,77) |
| Proverbs (Free inquiry)  n-total = 356 | 10.8 $\pm$2.5 (3 ,16) |
| Word-context (number of consecutively correct)  n-total = 262 | 25 $\pm$6.1 (7 , 38) |
| 20-questions (initial abstractions)  n-total = 360 | 30.4 $\pm$13.3 (2 , 60) |
| 20-questions (total asked questions)  n-total = 354 | 27.8 $\pm$7 (17 , 55) |
| 20-questions (total weighted achievement score)  n-total = 356 | 15 $\pm$2.7 (6 , 20) |
| RAVLT (T1-immediade recall)  n-total = 254 | 6 $\pm$1.6 (1 , 10) |
| RAVLT (T5-immediade recall)  n-total = 255 | 11.7 $\pm$2.3 (5 , 15) |
| RAVLT (total immediate recall)  n-total = 254 | 48.1 $\pm$9.2 (18 , 67) |
| RAVLT (delayed recall)  n-total = 255 | 9 $\pm$3.5 (0 , 15) |
| RAVLT (delayed correct recognition)  n-total = 251 | 12.7 $\pm$1.9 (6 , 15) |
| RAVLT (delayed false recognition)  n-total = 252 | 1.5 $\pm$1.8 (0 , 8) |
| Anxiety (State)  n-total = 466 | 30.6 $\pm$9.3 (20 , 71) |
| Anxiety (Trait)  n-total = 466 | 33.7 $\pm$9.4 (20 , 67) |
| NEO (N total)  n-total = 462 | 17.2 $\pm$7.4 (0 , 42) |
| NEO (E total)  n-total = 461 | 30.3 $\pm$5.8 (12 , 48) |
| NEO (O total)  n-total = 462 | 32 $\pm$5.9 (16 , 48) |
| NEO (A total)  n-total = 463 | 35 $\pm$5.7 (18 , 48) |
| NEO (C total)  n-total = 463 | 34.6 $\pm$7 (13 , 48) |
| **clinical sample** | **Participants** (n= 371, 200 male; 39 Sites) |
| Age (years) | 71.6 $\pm$7.4 (55 , 91) |
| Education (years) | 16.2 $\pm$2.5 (9 , 20) |
| Diagnosis [n] [SMC / EMCI / LMCI / AD] | 47 / 177 / 85 / 62 |
| MMSE | 28 (19 , 30)* |
| RAVLT (total immediate recall) | 36.6 $\pm$12. 75 (1 , 68) |

**Data are mean ± standard deviation (minimum-maximum), unless indicated otherwise.**

*** median (minimum-maximum).**

Abbreviations: BMI : body mass index; IQ : intelligence quotient; ANT : attention network task; CWI: color-word interference task; RAVLT : Rey auditory verbal learning task; NEO five factor inventory (N: neuroticism, E: extraversion, O: openness, A: agreeableness, C: conscientiousness); SMC: significant memory complaint; EMCI: early mild cognitive impairment; LMCI: late mild cognitive impairment; AD : Alzheimer’s disease.
